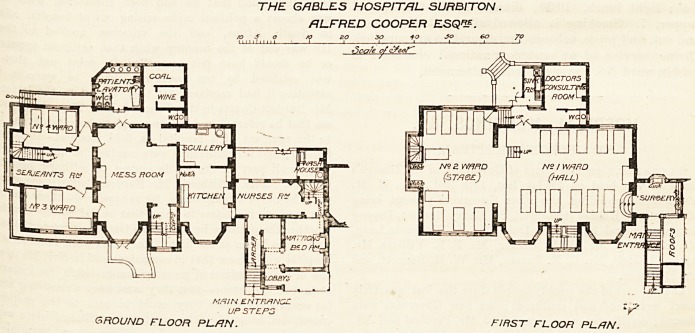# Hospital Construction

**Published:** 1900-07-21

**Authors:** 


### The Institutional Workshop.

### HOSPITAL CONSTRUCTION.

### THE GABLES HOSPITAL, SURBITON.

The conversion, by Mr. and Mrs. Alfred Cooper, of the model theatre in their grounds at Surbiton into a fully-equipped military hospital, is an event of historical importance. This is the first occasion on which private munificence and enterprise have been turned in such a manner to account, and it was not until the experiment had been completely tested that it received the benediction of the Army Medical Department. The original intention was to provide exclusively for the sick and wounded soldiers returning from South Africa on the "Princess of Wales" hospital ship, and it was at the express wish of the Princess of Wales herself that the offer of Mr. and Mrs. Cooper was accepted. It has since been found, however, that its scope was capable of extension, and that The Gables Hospital is able to afford relief to Netley, receiving, as a matter of fact, some of the most serious cases needing operations. The primary purpose will, of course, be adhered to, and beds will always be made available for sufferers arriving from the front on board the "Princess of Wales"; but any vacancies occurring during the intervals in her voyages are filled at the discretion of the authorities.

The hospital has a singularly pleasing exterior of the Gothic style, and it is so close to Surbiton railway station that patients can, if necessary, be carried in without difficulty. The patients are usually sent from Netley in an ambulance carriage, and are always met at the station by Mr. Cooper, and some of the medical staff; and a meal is served within ten minutes of their arrival.

There is accommodation for 27 beds, which are distributed in four wards. In No. 1 Ward, formerly the hall of the theatre, there are 11 beds. They are placed on either side of the hall, with three feet of space intervening, and between each is a locker for the personal belongings of the men. Meal tables, which can be slipped over the beds, are provided. In the centre of the ward are a number of prettily-arranged little tables, with draughts, and other games. There are seven luxurious easy chairs, a comfortable sofa, and a book-case full of novels, which are replenished every week. Pictures lent by a Royal Academician adorn the walls, and over the mantelpiece at the end of the ward fresh flowers are placed every morning, surrounding a fine portrait of H.R.H. the Princess of Wales. The ward can be heated by an open fire and by radiator hot-water pipes. In the corner, on the right hand side, in a recess by the window, behind a tastefully-arranged screen, is a wheel bath. Hot or cold water baths can thus be had at any time, and the patients can be wheeled back into bed. There are two Berkefeld filters in the ward, and an ample supply of spittoons.

At first a small room, now the surgery, was used as an operating theatre, but part of the stage, which is ward No. 2, has been made available for the purpose, the stage curtain being drawn. Here there are 10 beds. This ward, like the rest of the hospital, is splendidly lighted, and has three large plate-glass windows. After the cases are operated upon, they are placed in the beds close at hand. The operating table is of the usual height and length, and there is every convenience in the shape of mackintoshes, towels, bandages, receivers, hand-bowls for antiseptics, lockers for instruments, and pails for dressings. There are three large earthenware reservoirs for lotions, supplied by Messrs. Downs, with indiarubber tubing attachments, which are specially fitted for an operating theatre. The floor is stained wood, and the ward is warmed by radiator hot water pipes.

Adjoining ward No. 1 are a lavatory and the doctors' consulting-room. The medical staff consists of a house surgeon and five doctors, who see the cases every day. There is telephonic communication with the private house of each of the staff. A consulting and operating surgeon is also attached to the hospital.

GROUND FLOOR PLAN. FIRST FLOOR PLAN.

In the basement, which is light and airy throughout, there are two small wards, each with three beds. Between these is the sergeant's room. The mess-room is admirably appointed, and will seat thirty. In addition to the kitchen and scullery on the same level, there is a cloak-room for the use of the patients, a lavatory with four basins, with hot and cold supplies, and towels on rollers, a full-sized bath, and closets carefully detached. Provision is made in the basement for the matron, who has a room of her own, and for the four nurses and other members of the staff.

Outside the hospital there are many exceptional features of interest. The men sit on a balcony with a flight of steps overlooking an asphalte tennis court, surrounded by picturesque and well-timbered grounds. Beyond this, on the middle lawn, is a football field, and on the upper lawn a shooting range has been put up, which has been approved of by Colonel Phillips, the commanding officer at Kingston Barracks. For the amusement of the inmates a grand concert phonograph is used in the grounds, and in the chief ward a beautiful musical box, the gift of the Princess of Wales, plays soft music, and as the patients get better they are entertained each night. Five meals are given each day—Breakfast, 7.45; light lunch, 10.30; dinner, 12.45; tea, 4; and supper, 7. Smoking is allowed all day both indoors and out, and pipes, tobacco, and cigarettes are supplied. Divine service of an undenominational character is held in ward No. 1 every Sunday afternoon.

## Figures and Tables

**Figure f1:**